# Depicting corrosion-born defects in pipelines with combined neutron/γ ray backscatter: a biomimetic approach

**DOI:** 10.1038/s41598-020-58122-3

**Published:** 2020-01-30

**Authors:** M. Licata, M. D. Aspinall, M. Bandala, F. D. Cave, S. Conway, D. Gerta, H. M. O. Parker, N. J. Roberts, G. C. Taylor, M. J. Joyce

**Affiliations:** 1 Lancaster University, Engineering Department, Lancaster, UK; 2 Hybrid Instruments Ltd, Lancaster, UK; 3 The National Physical Laboratory, Teddington, Middlesex UK

**Keywords:** Mechanical engineering, Imaging techniques

## Abstract

The identification of corrosion, cracks and defects in pipelines used for transporting oil and gas can reduce the possibility of leaks, and consequently, it can limit the extent of an environmental disaster, public hazard and the associated financial impact of such events. Typically, corrosion in oil pipelines is measured with non-destructive ultrasonic or electromagnetic techniques, on the basis that corrosion and defects are often manifest as a change of thickness in the steel from which pipelines are made. However, such approaches are not practical for underground pipelines and their deployment can be complicated for the case of pipelines covered by insulation. In this paper, we present an innovative, non-destructive testing technique, which exploits the backscatter of a combination of fast-neutron and γ radiation from steel samples of a variety of thicknesses consistent with changes that might arise due to corrosion of a pipe wall. Our research demonstrates the potential to measure and characterise different steel thicknesses by detecting both the elastic, fast-neutron backscatter and the Compton-scattered γ radiations, simultaneously. Further, we demonstrate that the presence of insulation yields a consistent and separable influence on the experimental, wall-thickness measurements. The data from experimental measurements are supported by a comprehensive Monte Carlo computer simulation study.

## Introduction

The processes by which mechanical and electromagnetic waves are reflected by materials, are phenomena exploited by a variety of animal species for orientation, to procure food and for a great diversity of other purposes. A biomimetic relationship exists in this regard between the natural world and technological human achievements, exemplified on the one hand by the reliance of some species of mammals (predominantly bats) on ultrasound, with which to hunt, avoid predators and even to classify different types of plants^1–5^, and on the other by sonar^6^. The latter is central to a wide variety of non-destructive, industrial assessment techniques and a related international industry, such as the measurement of distance, density, porosity and imaging. Similar analogies exist for the case of reflected electromagnetic waves, e.g., sight, radar^7^ etc.

Amongst the first observations of the scattering of particles are those of Rutherford in his famous gold foil experiments (1908–1913). At the atomic scale, the reflections of waves and particles approach one another phenomenologically, and offer one of the founding scientific observations supporting wave-particle duality. In this regard, neutron scattering and its applications are perhaps amongst the most remarkable and tangible exemplars of quantum-mechanics. As to whether a scattering process is elastic or inelastic is inferred by the corresponding isotopic cross section, calculated on the basis of neutrons affording properties of complex plane waves. Below the MeV range in energy, the radiation wavelength is much greater than the range of the strong nuclear force (of the order of femtometres) that is responsible for scattering from a single nucleus. This renders neutron scattering, according to the first-order Born approximation, isotropic. Similarly, the same approximation, is applied in radar^8,9^.

Scattered radiation is exploited in several non-destructive assessments. Electron backscatter is the principle of the scanning electron microscope^10^, muon scattering tomography has been tested for nuclear reactor core imaging^11^, monitoring volcanic activity^12^ and the detection of chambers in pyramids^13^. X-ray backscatter radiography (BCT) has numerous applications in safety and security inspection, particularly for the detection of dangerous materials and border inspections^14^, biomedical science^15^, engineering and industry, such as the oil and gas sector and aerospace^16–18^. The advantage of backscatter radiography and tomography is that they allow the investigation of items that cannot be scanned with the most widely-used axial computerized tomography (CT), due to either the large size of the item and inability for it to be moved, or because the transmission image data is not interpreted easily. Elastic neutron scattering is usually used to assess water content, porosity and hydrogen fraction in rocks^19,20^, and to identify the presence and assess the level of water, gas, wax, paraffin and/or defects in pipelines^21–25^. Elastic scattering cross sections and the loss of energy after a single scattering event are fundamental to many non-destructive, fast-neutron backscatter techniques. In comparison with X- and γ rays, fast neutrons penetrate deeper into high-Z matter, due to their lack of charge, and thus high-density materials can be investigated. X- and γ-ray backscatter is the result of the Compton effect; its probability depends on the electron density of the material. On the one hand, this makes X- and γ rays particularly suitable for the investigation of heavy metals, but, on the other, limits the extent to which they can be used to probe relatively thick material samples, because high-Z media attenuate them significantly.

In this research, the backscatter of fast neutrons and γ rays is used to identify different carbon-steel thicknesses. Our approach is to exploit and possibly combine two different imaging techniques, performed *real-time*, simultaneously with a single source-detection system. Defects in steels, as well as corrosion and rust, produce a variation of the mean density and of the thickness of the steel comprising the pipe wall; therefore the backscattered neutron and γ-ray flux induced by a radiation beam changes. Pipelines are subject to several types of corrosion. It can be internal or external. The former is mainly due to galvanic corrosion, microbiological reasons, stray currents and selective seam weld corrosion, and is often exacerbated by the presence of crude oil, hydrogen sulphide, carbon dioxide, various natural gases, vapours and water^26,27^; the latter is caused by a pipeline carrying a corrosive commodity, low-pH aqueous media and erosion. The quantification of corrosion-born defects is particularly important for industries that rely on many thousands of kilometres of pipelines for transporting resources and also to inform estimates of resources required to replace compromised pipeline parts. Statistical results show that, in the U.S., the U.K and Europe from 2010 to 2015, 24% of failures in gas pipelines and 25% in oil pipelines, were due to corrosion. Failures in pipelines can cause economic losses, environmental pollution, injuries and casualties. Failure frequencies of oil pipelines range from 0.4 to 0.6 times/kkm/yr in the U.S. whilst gas pipelines failure ranges from 0.04 to 0.14 times/kkm/yr^28^. The primary form of corrosion is pitting, and numerous mathematical and numerical models have been studied to predict the specific corrosion rate of this^29–31^. The evolution of corrosion pitting over time is considered constant, universally, with a linear damage-velocity rate. Corrosion depth depends on the time *t* according to *αt*^*β*^, with *α* and *β* constants that depend on the system, the pipeline environment and type of corrosion^32^. These constants are evaluated from in-field measurements and come from fits to corresponding data. These measurements to yield such data, as well as the experimental estimation of corrosion rates, are usually carried out with periodical in-line inspections.

Most in-line inspections are carried out by means of Pipelines Inspection Gauges (PIGs) based on ultrasound^33–35^. However, ultrasound requires a coupling medium, such as oil, and therefore these inspections are mainly applied to liquid lines and not for gas pipelines. Our approach is, to some extent, complimentary to the ultrasound technology, since it probes the pipeline from outside, and demonstrates potential to be able to measure different steel thicknesses in the presence of insulation (i.e., pipelines covered by layers of concrete and polyethylene). We present a radiography/tomography system in which a mixed radiation field (comprising neutron and γ), produced by a californium source (^252^Cf), is collimated with a combination of lead and polyethylene to produce a *pencil*-*like* beam of probing radiation (it is anticipated that a portable neutron generator could be used in place of an isotopic source if necessary). Finally, this is directed toward the steel under consideration. The induced fast-neutron and γ-ray backscattered flux has been measured with four organic liquid scintillation detectors with the collimator described above, to constitute a prototype connected to a mixed-field analyser (MFA), providing *real-time*, digital, pulse-shape discrimination (PSD)^36^, to yield transistor-transistor logic (TTL) signals that are retained and recorded with a digital counter. A mechanical rig enables the source-collimator arrangement to raster across a sample to afford scans of each steel sample under inspection. This system, as described, enables combined, simultaneous, fast neutron and γ-ray backscatter imaging.

A variety of steel thicknesses have been investigated with the influence of insulation replicated by placing a layer of concrete and high density polyethylene (both of 1-cm thickness) above the steel. The novelty of combining different imaging modalities leads to improved discrimination of contrasting material thicknesses, particularly when layers of concrete are used to replicate insulation of this type covering a pipe. The contrasting properties of neutrons and γ rays, allows a fine depiction of pits in pipelines, from both a qualitative and a quantitative point of view. By analogy with the natural world, the collimated beam of neutrons and γ rays can be compared to the chirp generated from bats, which is a superposition of different ultrasound wavelengths, read simultaneously after their interaction with the surroundings, by the same ear-detector. Furthermore, the sensitivity modulation of the sensory system to their own self-vocalised, ultrasonic pulses in some bat species^37^, which is understood to enable the return to be better isolated, constitutes an evolutionary feature comparable to the collimator in our system. This, albeit infinitely more crudely, blinds the scintillation detectors from the direct, un-scattered component that would otherwise perturb the sensitivity of the system to the backscattered component. Similarly, some nocturnal moths have evolved wings that absorb ultrasound, suppressing the echo that the bat receives and rendering the moths invisible to these predators^38,39^. It appears likely that the nature of the backscattered return provides the bat with a significant amount of information as to the type and extent of material around them, as to whether it is solid, diffuse, moving, stationary, etc.

## Results

### Detection system and neutron-γ collimator

**Figure 1:**
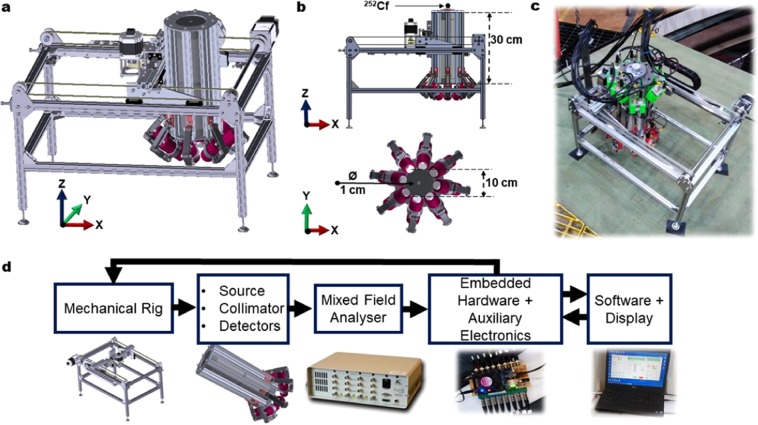
System assembly. (**a)** A 3-dimension CAD design of mechanical rig, collimator and detectors, designed, assembled and demonstrated in this research. **(b)** The elevation (top) and plan (bottom) CAD views of the system, with a focus on the collimator-detector combination. The collimator is made of layers of lead and polyethylene, height: 30 cm, external diameter: 10 cm and internal diameter of the pinhole: 1 cm. The radiation source is placed above the collimator, concentrically with the pinhole. **(c)** A photograph of the system built, deployed and used at the low-scatter facility of the National Physical Laboratory. **(d)** A sequential schematic of the experimental set-up: the mechanical rig, controlled remotely by software running on a laptop, drives the system comprising the source, collimator and detectors. The detectors are connected to a multiple-channel, mixed-field analyser, which digitises the detected events, separates the neutron and γ-ray signals and sends a corresponding transistor-transistor logic signal for each event to an embedded digital counter that is linked by Ethernet to a computer. The entire system is coordinated and controlled by a graphical user interface.

The neutron and γ-ray flux backscattered from a variety of different steel slabs was measured with a system designed for this purpose, comprising: a mechanical rig, collimator and an array of small, organic scintillation detectors (Scionix, Netherlands) which were connected to a multiple-channel, mixed-field analyzer (Hybrid Instruments Ltd.) and to a bespoke embedded control system. Measurements were carried out at the low-scatter neutron facility at the National Physical Laboratory (NPL), in Teddington, London, UK. The rig-source-collimator-detector system consists of an aluminum frame in which two stepper motors have been configured to afford the system two degrees of freedom (in the X-Y plane, Fig. 1a,b). The collimator is designed to constrain the neutron and γ radiation generated by the ^252^Cf to a defined area of interest of the steel sample, comprising cylinders of high-density polyethylene (HDPE) and lead (Pb).

**Figure 2:**
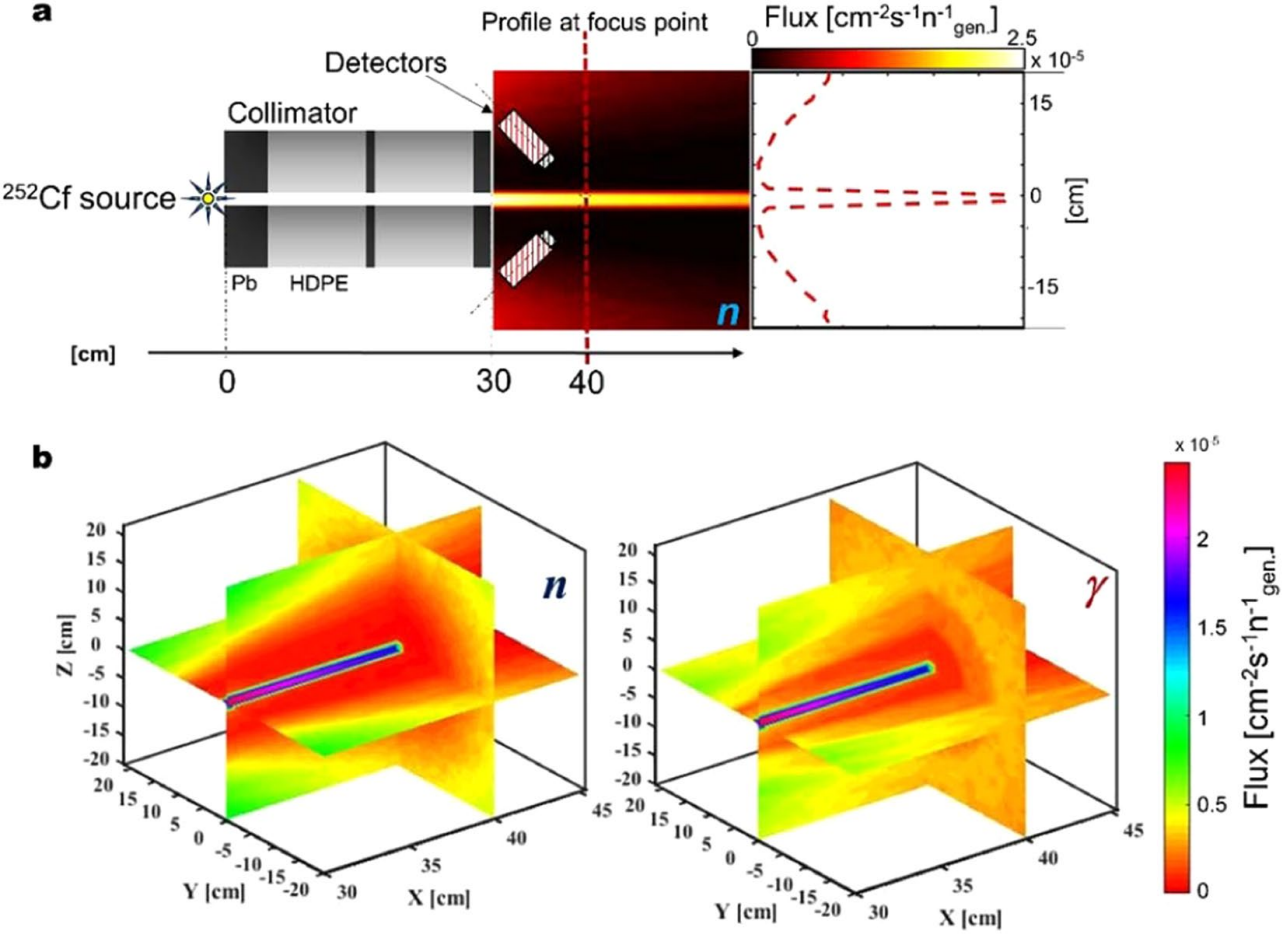
Collimator modeling. (**a)** A schematic representation in elevation of the source-collimator-detector arrangement (from left to right) combined to demonstrate the relative, quantitative significance of the fast neutron flux throughout. The colour heatmap depicts the neutron flux leaving the collimator, whereas the plot on the right presents a quantitative measure of the same flux along the dotted red line passing through the detector focus point. **(b)** An MCNP6, 3-dimensional quantitative illustration of both fast neutron (left) and γ-ray flux after the collimator aperture, demonstrating the relatively small component of the direct field impinging on the detectors, consistent with the fundamental concept explored in this research.

Organic liquid scintillation detectors of type EJ-301^40^ are secured in a fixed angle of orientation with adjustable arms. Up to eight detectors can be used in the geometrical configuration shown in Fig. 1. However, it has been found that four detectors enable the total geometric efficiency to be optimised, i.e., maximising the area of the hemisphere subtended by the detectors. The position of the radiation-sensitive liquid-volume in the detectors has been configured to be in a region of the space below the collimator sheltered from the radiation flux that streams from the 1-cm diameter, collimator pinhole (Figs. 1b and 2a). With this particular approach, the detectors are isolated from the incident flux but, at the same time, they are positioned sympathetically with the trajectory of the backscattered flux generated at the focus of the radiation beam collimated by the pinhole. The beam-and-detector focus-area is the point where the radiation beam reaches the surface of a given sample, and it is located approximately 10 cm after the collimator aperture (Fig. 2a).

An extensive Monte Carlo computer simulation study was performed in order to identify the best collimator geometry and detector position. MCNP6^41,42^ (Monte Carlo N-Particle transport code), developed in Los Alamos National Laboratory^43^, is the tool used to model the experiment and compute the simulations. The results are presented in Fig. 2a,b. Neutron and γ-ray flux are shown in 3-dimensions for the X-Z, X-Y and Y-Z planes. In particular, the plots show the flux beyond the collimator aperture, with the Y-Z plane modelled at X = 40 cm, i.e., approximating to the radiation focus at the sample. Detectors are located at *circa* 5 cm from the beam, in the position of minimum flux.

### Calibration

**Figure 3:**
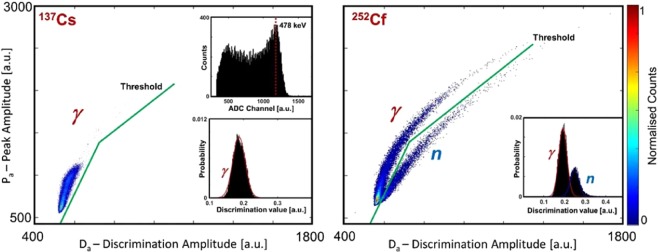
Pulse shape discrimination. Left: A scatter plot (peak amplitude versus discrimination amplitude) of the γ radiation generated by ^137^Cs with an EJ-301 detector in this research. The peak and discrimination amplitudes are, respectively, the maximum signal amplitude and the signal magnitude measured after a fixed discrimination-time. The top-inner plot is the Compton spectrum when an EJ-301 is exposed to γ rays from ^137^Cs, whereas the bottom-inner histogram is the discrimination value of the γ-event produced with ^137^Cs. Right: A scatter plot obtained with an EJ-301 scintillation detector exposed to ^252^Cf in this research. The upper plume corresponds to the γ component of the mixed field produced by ^252^Cf, whereas the lower plume corresponds to the neutron component. The inner histogram (lower-right) shows the discrimination values for n-γ events produced by ^252^Cf. Signals have been normalised and their baseline removed. The discrimination data have been fitted (red lines) with Gaussians.

The EJ-301 detectors were calibrated prior to the backscatter measurements by setting the high-voltage of the photomultiplier of each detector, to yield a balanced response across all four units, and the pulse-shape discrimination parameters were adjusted via the MFA to optimise the discrimination between neutrons and γ rays. Using a 17 MBq ^137^Cs source, the voltage of the scintillator photomultiplier was adjusted to align the caesium Compton edges (at ~478 keV) to the same ADC channel (Fig. 3, left, inner plot on the top). The use of the ^137^Cs source ensures a response to γ rays of a single energy (662 keV), thus making possible to set the discrimination threshold on each individual scintillator, using only the γ plume (Fig. 3, scatter plot on the left). Pulse-shape discrimination is performed by the MFA via a pulse gradient analysis algorithm^44^. The discrimination value (i.e., the ratio between discrimination amplitude and signal peak amplitude) of each γ event generated by the aforementioned caesium source was also calculated. The results (Fig. 3 left, inner plot on the bottom) show the presence of a single peak, consistent with only γ radiation being present for the case of the ^137^Cs source. Subsequently, ^252^Cf was used in order to verify the *n-*γ response of the scintillators and to verify the PSD threshold settings. Two separate plumes were observed (Fig. 3, scatter plot on the right) consistent with an appropriate PSD setting; two peaks are also observed when the discrimination value of the mixed neutron/γ field events is plotted as a histogram.

### Neutron-γ backscatter

**Figure 4:**
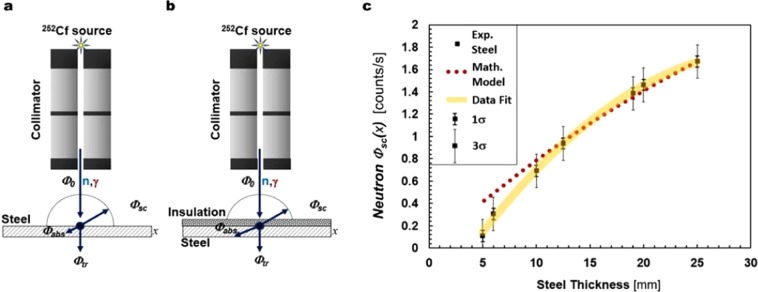
Mathematical relationship. (**a,b)** Schematic illustrations of the mathematical model developed in this research for steel and a combination of steel and insulation. **(c)** Backscattered neutron flux versus thickness of bare steel. The yellow band represents a second-order polynomial fit to the data, of 1σ spread. The theoretical model is represented by the red dotted line. Σ_tot_ has been estimated using the tabulated neutron cross sections from ENDF/B-VII.1 of the National Nuclear Data Center^46^.

When a narrow, collimated radiation field hits a material, part of its radiation is transmitted, part is absorbed and part is backscattered (Fig. 4a). The flux of backscattered radiation, as a function of the material thickness, is related closely to the linear attenuation law,

1 $${\Phi }_{tr}(x)={\Phi }_{0}{e}^{-{\rm{\mu }}x}$$

where ${\Phi }_{tr}$ is the transmitted component for a thickness *x*, of the initial flux ${\Phi }_{0}$, μ is the linear attenuation coefficient for a field comprised of γ rays, whilst it corresponds to the total macroscopic cross section (Σ_tot_) in radiation fields constituted by neutrons^45^. The backscattered flux can be expressed as,

2 $${\Phi }_{sc}={\Phi }_{0}-{\Phi }_{abs}-{\Phi }_{tr},$$

where ${\Phi }_{abs}$ is the absorbed component of the radiation flux, for a thickness *x*. Thus, the scattered flux is given by,

3 $${\Phi }_{sc}(x)={\Phi }_{0}(1-{e}^{-{\Sigma }_{tot}x})-{\Phi }_{abs}(x)$$

If the radiation encounters a combination of different materials (see example depicted in Fig. 4b), whilst the physical principle is the same, the mathematical model becomes more sophisticated because both scattered and absorbed components derive from the superposition of effects from each of the different compounds. Figure 4c presents the measured, experimental backscattered neutron flux as a function of the steel thickness measured in this research; the backscattered neutron flux was assumed to be isotropic but this is not the case for γ rays, since Compton scattering is not isotropic. These experimental data are compared with the mathematical model presented in Eq. (3). Experimental results are presented with both ±1σ and ±3σ standard deviation and demonstrate consistency with the model given in Eq. (3).

**Figure 5:**
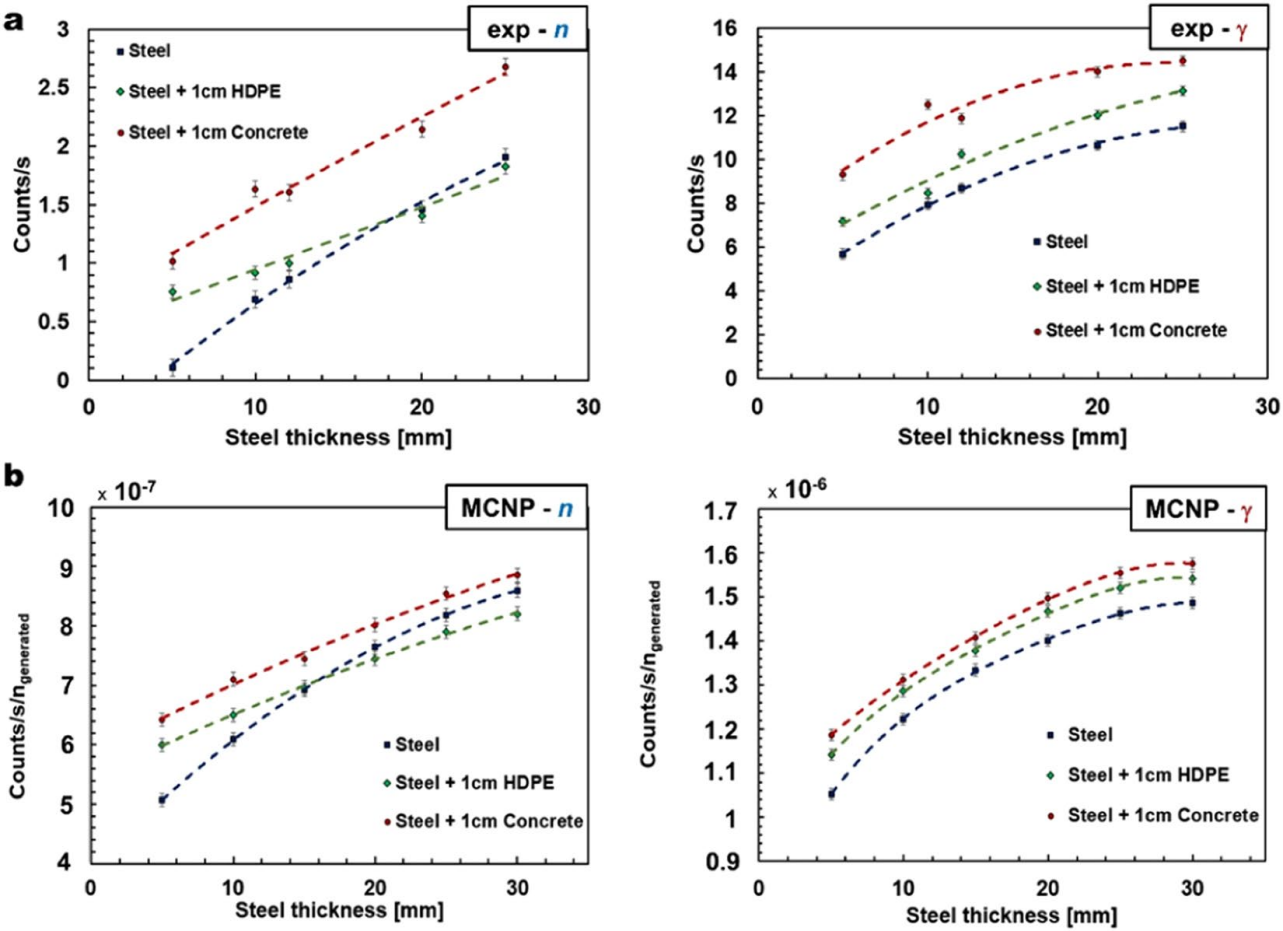
Experimental and simulation results. (**a)** Experimental results for neutron (left) and γ-ray (right) backscatter in terms of counts per second versus steel thickness, for bare steel (dark blue symbols), steel with 1-cm thickness polyethylene insulation (green symbols) and steel with 1-cm thick concrete as insulation (red symbols). **(b)** Results of Monte Carlo simulations performed with MCNP6; neutron (left) and γ-ray (right) backscatter in terms of normalised counts per second, per neutron generated in the Monte Carlo simulation, versus steel thickness, for bare steel (dark blue symbols), steel with 1-cm thickness polyethylene as insulation (green symbols) and steel with 1-cm thickness concrete as insulation (red symbols).

The flux of backscattered neutron and γ rays are presented in Fig. 5a, as a function of the steel thickness in the presence of a 1-cm thick layer of high-density polyethylene and 1-cm thick layer of concrete, to illustrate the effect of insulation on the technique. The reflected neutrons and γ rays were measured over a period of 20 minutes for each individual sample of steel, for a total of 1 hour per slab given the three cases as follows: bare steel; steel and polyethylene; and steel and concrete. The results from these measurements are compared qualitatively with the corresponding results from MCNP6 simulations (Fig. 5b) obtained prior to the experiment. In this case, uncertainties are presented as ±1σ and the fit to the data is a second-order polynomial function.

The possibility of γ rays arising from inelastic neutron reactions exists but this is anticipated to be small for the energy of the neutrons from ^252^Cf and therefore this has not been accounted for in this research. If sources with harder neutron spectra are considered for this application, such as AmBe or D-T generators, then this influence would need to be quantified.

### Combined n-γ backscattered tomography: a case study

**Figure 6:**
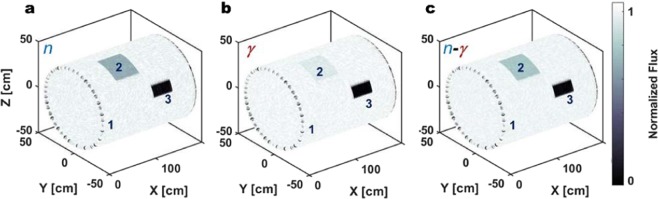
Neutron-γ imaging. Fast neutron **(a)**, γ-ray **(b)** and combined n-γ **(c)** backscatter tomography of a 25-mm thick pipeline of 40 cm radius. The different regions of pipe wall thickness are identified as per: region 1 denotes the unadulterated 25-mm thick region of pipe, 2 and 3 indicate the 20-mm and 5-mm thick regions included to illustrate contrasting degrees of corrosion severity, respectively. The backscattered flux has been normalised and plotted using greyscale, as is used by convention in tomography studies.

For the case of neutron and γ-ray backscatter from a sample comprising steel and concrete (included by way of insulation), a relevant scenario is that of a steel pipeline of 25-mm wall thickness, insulated with concrete and which is to be scanned to identify regions of corrosion. Using the experimental data of Fig. 5a (red dotted curves) of such a 25-mm pipeline, an exemplar pipe tomography study has been conceived and is presented in Fig. 6. Two different regions of different thicknesses have been inserted deliberately, and positioned randomly in the pipeline wall to emulate regions of corrosion. The first region is 5 mm thick and the second region 20 mm (Fig. 6a, denoted by numbers 2 and 3). The experimental backscattered flux from each of these features has been reproduced and imaged, together with the backscattered flux of the original 25-mm thick pipe. Figures 6a–c show the fast neutron backscatter tomography (FN-BCT), the γ-BCT and the combined *n-γ* BCT, respectively, for this case. The 5- and 20-mm regions, that render the steel respectively 20- and 5-mm thick, are clearly discernible using fast neutrons, whereas the 5-mm pit-area is not easily-discernible scanning the pipe with γ rays in isolation. Combining the two different imaging modalities, it is still possible to identify the two areas of reduced thickness. However, this scenario can arise in reverse, that is, depending on which materials are scanned, the neutron tomography data that results might mislead, in contrast to the γ-ray case, as explained in previous work for transmission tomography^47,48^.

### System sensitivity

The minimum time necessary to discern between different thicknesses of steel with and without insulation, can be correlated with the sensitivity of the system. Hypothetically, when the number of accumulated counts from the detectors is plotted as a function of time, backscattered counts arising from contrasting wall thicknesses will have similarly-contrasting gradients or count rates. In this particular circumstance, after a given observation time *t* has elapsed, the error (*σ*) on the counted number of events (*N*) is the square root (*σ* = *√N*), assuming Poisson statistics. Equation 4 describes the minimum time needed to differentiate two different thicknesses of the same material, within a sensitivity of *n*_*σ*_ = 1, 2, 3… standard deviations,

4 $$t\ge {\left(\frac{{n}_{\sigma }[\sqrt{tg\alpha }+\sqrt{tg\beta }]}{tg\beta -tg\alpha }\right)}^{2}\equiv {\left(\frac{{n}_{\sigma }[\sqrt{{N}_{1}}+\sqrt{{N}_{2}}]}{{N}_{2}-{N}_{1}}\right)}^{2}$$

where *N*_1_ and *N*_2_ are the counts, at a given time *t*, of the two thicknesses.

**Figure 7:**
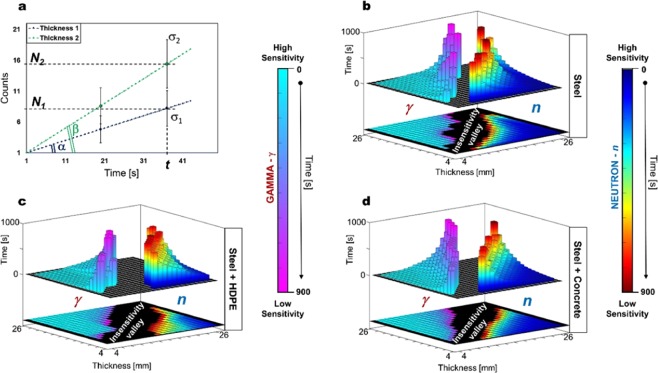
Sensitivity matrix. (**a)** Qualitative and quantitative example of the minimum time needed to separate two different backscattering counting rates with different standard deviations. **(b–d)** System sensitivity matrices for γ rays (colour map *light blue-to-magenta* type) and neutrons (colour map *blue-to-red*) regarding steel (**b**), steel and polyethylene (**c**) and steel and concrete (**d**). The colour scale levels in the matrices, coupled with the height of the histograms, indicate quantitatively and qualitatively the time. Times above 900 seconds are cut and plotted in black (depicted by the valleys between the neutron and γ-ray histograms).

An illustrative example with respect to this sensitivity formulism is given in Fig. 7a: Here, Eq. 4 has been used to construct a sensitivity matrix for neutrons and γ rays, for the system tested with bare steel (Fig. 7b), steel with polyethylene (Fig. 7c) and steel with concrete (Fig. 7d). The left side of the matrices in Fig. 7b–d corresponds to γ rays and the right to neutrons. Two different colour maps for γ rays and neutrons have been used to separate the sensitivity of each, and a 900-second cut-off on the minimum measurement time necessary to distinguish two thicknesses has been set deliberately, on the basis of what is anticipated to be an acceptable limit in the field. Generally speaking, times lower than this value allow the discrimination of difference in thicknesses from 5 mm and upwards. For the identification of possible pits of less than 5 mm depth, the measurement time required increases exponentially, at which point this technique starts to become impractical. The sensitivity matrix presented here has been calculated with the experimental data presented in Fig. 5a, using a ^252^Cf source with an emission rate of 8.7 × 10^6^ neutrons/second into 4π. The sensitivity can be improved and thus the experimental exposure time reduced using a source of higher activity. Alternatively, shielding the detectors in order to reduce false-negative scattering events due to background and cross-talk between detectors could also improve the sensitivity.

## Discussion

The results presented in this paper demonstrate that it is possible to discern different thicknesses of steel slab with a combination of fast neutron and γ-ray backscattering. Our research was carried out using organic liquid scintillators, although a diversity of organic scintillators exists which could be similarly applied; for example stilbene might constitute a valid alternative to the EJ-301 used in this research if a liquid scintillant is not desirable in the application field. Organic scintillators, coupled with the real-time PSD system used in this research, are particularly suitable because their detection efficiency falls sharply for energies below ~1 MeV for neutrons and below 200 keV for γ rays. The elastic scattering cross sections for neutrons in the range 1–10 MeV, are of the same order of magnitude for the majority of elements (between 1 barn and 10 barns). However, since the neutron energy loss after an elastic collision is far greater for low-atomic number materials, incident fast neutrons can fall below the detection energy threshold of the scintillator detectors when scattered. This fact, for example, explains the difference in slope in the responses for steel and steel-polyethylene for neutrons in Fig. 5a,b, since HDPE is rich in hydrogen relative to concrete, and thus moderates neutrons more effectively. For small thicknesses (i.e., ≤15 mm), the polyethylene-induced backscattering is higher than that measured with bare steel. As the steel thickness is increased, the number of elastic neutron collisions also increases; therefore, before reaching the detector, the backscattered neutrons pass through an extra thickness of polyethylene, accruing a higher probability of falling below the energy threshold for detection, and thus reducing the proportion of the backscattered component that is detected. Conversely, this does not occur for γ rays because Compton scattering depends on the atomic number of the material, which is relatively low for polyethylene compared to that of steel, and consequently, the gradient of the γ-ray backscatter count dependence with thickness is similar for all three sample arrangements. Our results are consistent with what is predicted on a qualitative basis by the MCNP6 simulations for both neutrons and γ rays. The mathematical relationship elaborated for neutrons overlaps the neutron experimental data for the case of bare-steel, in the range 6–25 mm, when results are presented within ±3σ from the mean.

The novelty of this research lies, *in primis*, in the demonstration of a non-destructive imaging alternative to the widespread modality of X-ray computed tomography. Moreover, this technique comprises the parallel application of both neutrons and γ rays, leading to three different final illustrations of a given sampler (i.e., n-, γ- and n-γ-BCT). Read together, these yield a more comprehensive and faster representation of the inner structure of steel and possibly, other materials. Our research highlights and confirms the potential of combining different imaging modalities. Not only does this technique have applications in an engineering context, but it may also have potential in wider materials science applications such as for quality assessments of metals and materials, and also in a wide range of different scenarios, ranging from the medical field (as proposed by^49^) to safety and security inspections, and particularly where *in situ* examinations are required. This research not only highlights the benefit of combining the effects of contrasting reflection phenomena for technological requirements, such as non-intrusive corrosion assessment; it also illustrates the significant potential that can accrue from our primitive mimicry of sensing modalities that have evolved for analogous requirements in the natural world.

## Methods

### The mechanical rig

The rig frame is made from an assembly of 20 × 20 mm aluminium extrusions, it has dimensions 600 × 400 × 340 mm (L × W × H). The collimator sits within the aluminium frame and is mounted to four guide rails, two in both the *X-* and *Y*-axis, respectively. This gives the collimator the freedom of movement in the X-Y plane, actuated using a stepper motor and pulley system. A symmetrical array of detectors are mounted to a cylindrical assembly using Go-Pro arms. The assembly is made from eight aluminium extrusions around 360° with aluminium plates top and bottom; the cylindrical components of the collimator itself sit flush within the extrusion assembly. The entire assembly has a height of 311 mm and a diameter of 140 mm. On the top plate of the assembly, a bespoke 3D-printed component is mounted to house the isotopic source directly above the collimator void. The rig is controlled using an Arduino® microcontroller board which interfaces with the user’s device via USB. All electronic components on the rig are controlled by the Arduino® which receives commands from the user. The user specifies coordinates relating to a position in the X-Y plane, the Arduino® then handles the calculation necessary to get to the desired position. Limit switches at the end of each axis are used to calibrate the positioning of the collimator assembly as well as a fail-safe to prevent it from driving off the rails.

### Control system and counter

The acquisition system consists of a printed circuit board (PCB) that contains an Intel Cyclone V FPGA/ARM Processor system. A set of sixteen 32-bit transistor-transistor-logic (TTL) compatible counters were configured on the FPGA. The system uses a 50 MHz clock signal and Phase-locked Loop (PLL) logic allowing pulses of less than 1 μs width to be detected. The 4-channel MFA produces TTL signals from separate output ports dependent on whether a neutron or a γ ray has been detected. The 4 γ-ray outputs and the 4 neutron outputs from the MFA were connected up to the aforementioned 16-channel counter (thus up to 8 detectors can be used: 8 channels for neutron detection and 8 channels for γ-ray detection). The Cyclone V ARM Processor system runs an embedded version of Linux capable of interfacing to the described logic in the FPGA part of the integrated circuit. A bespoke program developed in C++ was written to monitor the status of the 16-channel counter in real time. This monitor program utilised the TCP/IP Protocol to send data to a PC at a specified frequency. The counters can be set to either count-rate mode or cumulative mode for calibration and measurement operations, respectively. A bespoke program was written in C# for a PC, with an accompanying Graphical User Interface (GUI). This program has the ability to configure the aforementioned counter modes via the TCP/IP Protocol. This application also creates files to log all the information received from the FPGA board. Additionally, the application controls the actuators of the mechanical rig through the pre-configured serial port.

### The National Physical Laboratory low-scatter neutron metrology facility

It is located at the National Physical Laboratory in Teddington, London, UK. The room is 23 m long, 17 m wide and 18 m high. Walls are shielded by approximately 1 m of concrete^50^. The measurements in this research were carried out on a ground-elevated mobile platform, known as the pit-circle, roughly 6 m high. This particular platform can be accessed via a low-density, mobile, walkway.

### Radiation sources

A ^252^Cf source with an emission rate of 8.727 × 10^6^ neutrons/second (±0.6% at 1σ) and an approximate activity of 76 MBq, has been used for this research. The source anisotropy factor in the position with which the source was used, is 1.022. The source is encapsulated in a 1-cm diameter cylinder of stainless steel. The γ source used for the detector calibration was ^137^Cs, with an activity of 17 MBq.

### Detector stability, flux evaluations and background level

The whole experimental set-up was assembled approximately 20 hours prior to the start of the experimental measurements. Detectors, mixed field analyzer, electronics, embedded hardware and control software were turned on and their functioning monitored and verified. The stability of the detectors, with and without neutron and γ irradiation, was also demonstrated both in the laboratory at Lancaster University and at the National Physical Laboratory. Within 48 hours, during the preliminary tests carried out at Lancaster University, the counting rate of the detectors were observed to be constant over elapsed time. The baseline level of neutron and γ-ray background at the low-scatter facility was measured, as well as the background level induced by the ^252^Cf source in the room. Albeit being a low-scatter facility, a low-level of background is present due to the interaction of the radiation with air, room walls, laboratory and experimental components. This background was subtracted from the readings of the detectors when performing the data analysis. Finally, the mixed radiation flux from the collimator (Φ_0_, see Eq. 3) was evaluated carefully for each individual detector. This value has been 10 times higher than the value of the background induced by the californium source and measured by the detectors when placed in the position hidden by the collimator (see, for instance, Figs. 1b and 2a), confirming experimentally the validity of the simulations and collimator function.

### MCNP6 simulation

The final design of the collimator is the result of a detailed MCNP6 simulation study that has been performed in order to optimise the system geometry, materials and system characteristics. The collimator is a cylinder of 10-cm diameter and 30-cm length. It has a 1-cm diameter pinhole which allows the passage of the mixed-field radiation produced by the aforementioned radiation source. Three layers of lead (respectively of 3 cm, 1 cm and 2 cm) and two layers of high-density polyethylene (12 cm thickness each) shield the detectors from the radiation emitted by the source. The experiment carried out has been modelled as accurately as possible with MCNP6 simulations. Six steel thicknesses (from 5 mm to 30 mm) of a pipeline have been reproduced in this way. Neutrons and γ rays for scattered events from the pipeline wall have been tallied simulating the EJ-301 detectors and its liquid scintillant. The collimator and ^252^Cf source have also been modelled in the experiment-simulation. The steel (0.3% carbon component), concrete (Hanford type, dry) and polyethylene model details are listed in the Radiation Portal Monitor Project, Compendium of Material Composition Data for Radiation Transport Modelling^51^.

## Data Availability

The datasets generated and analysed during the current study are available from the corresponding author on reasonable request.
